# Case Report: Subacute combined degeneration misdiagnosed as a primary affective disorder: diagnostic pitfalls and clinical red flags

**DOI:** 10.3389/fpsyt.2026.1811233

**Published:** 2026-06-18

**Authors:** Yirui Dai, Yan Wang, Yuanyuan Chen, Jinfang Li, Xinyu Jia, Dongbin Cai, Guixiong Zhang, Haihong Guo, Songjiao Wei, Jianjun Wang, Xiude Qin

**Affiliations:** 1 The Fourth Clinical Medical College, Guangzhou University of Chinese Medicine, Shenzhen, China; 2 Department of Neurology and Psychology, Shenzhen Traditional Chinese Medicine Hospital, Shenzhen, China

**Keywords:** misdiagnosis, neuropsychiatry, somatic symptom disorder, spinal cord diseases, subacute combined degeneration, vitamin B12 deficiency

## Abstract

**Background:**

Subacute combined degeneration (SCD) of the spinal cord is a progressive neurological disorder caused by vitamin B12 deficiency. When initial symptoms present as nonspecific fatigue, dizziness, and affective distress, the condition is frequently misattributed to primary psychiatric disorders. This “diagnostic overshadowing” leads to critical delays in administering definitive therapy, risking irreversible axonal damage.

**Case presentation:**

A 39-year-old male presented with a nine-month history of progressive paresthesia, dizziness, and emotional distress. Initial medical workups (including brain CT and EEG) were unremarkable, leading to a diagnosis of Somatic Symptom Disorder and anxiety. Despite treatment with multiple psychotropic agents, his condition deteriorated, eventually manifesting as lower limb weakness and significant gait ataxia. Subsequent evaluation at a tertiary center revealed absent patellar reflexes and impaired vibratory sensation. Psychometric testing (SCL-90) showed a high somatization factor (3.00). Investigations confirmed a critically low serum vitamin B12 level (<37 pmol/L) and characteristic longitudinally extensive T2-weighted hyperintensities in the dorsal columns from C3 to T12. Following intramuscular and oral vitamin B12 replacement, supplemented by escitalopram for residual distress, the patient achieved significant recovery. At five-month follow-up, he was fully ambulatory with near-normalization of psychometric scores.

**Conclusion:**

This case illustrates the risk of misinterpreting organic neurological deficits as psychogenic somatization. A discrepancy between subjective psychiatric complaints and emerging objective neurological signs-such as absent reflexes or ataxia-should trigger an immediate search for underlying organic etiologies and the initiation of targeted treatment.

## Introduction

Subacute combined degeneration (SCD) of the spinal cord is a progressive neurological complication caused by a deficiency of vitamin B12 (1, 2). Pathologically, it is characterized by the demyelination of the dorsal and lateral columns of the spinal cord, resulting in a classic clinical triad of sensory ataxia, deep sensation deficit, and pyramidal weakness (3, 4). However, the clinical presentation of SCD is often heterogeneous. Beyond these core sensorimotor deficits, the deficiency can manifest with a broad spectrum of neuropsychiatric symptoms, ranging from subtle cognitive changes and irritability to severe depression, anxiety, and psychosis (5, 6).

This variability poses a significant challenge, particularly in the early stages of the disease when objective neurological signs may be subtle or absent (7, 8). The diagnostic complexity is compounded when psychiatric symptoms dominate the initial clinical picture—a phenomenon known as diagnostic overshadowing (9). In such scenarios, patients are at high risk of being misdiagnosed with primary psychiatric conditions (5, 6), such as Somatic Symptom Disorder or Generalized Anxiety Disorder. Such misclassification leads to critical delays in initiating vitamin B12 replacement, a time-sensitive window during which reversible demyelination can progress to irreversible axonal degeneration and permanent disability (2, 10).

We present a case of SCD in which evolving neurological symptoms were initially misattributed to a psychiatric disorder. This report illustrates the specific clinical and radiological “red flags”—including the characteristic “inverted V sign” on MRI—that distinguish organic neuro-metabolic disease from functional illness (11, 12). Our objective is to reinforce the necessity of a systematic diagnostic framework, emphasizing that in patients with mixed neuropsychiatric presentations, persistent somatic complaints warrant a rigorous exclusion of organic etiologies to ensure timely intervention.

## Case presentation

### History of present illness

A 39-year-old previously healthy male presented with a nine-month history of progressive fatigue, dizziness, and unsteady gait. Symptom onset was insidious, initially characterized by numbness in both upper limbs, a sensation of heaviness in the head, depressed mood, insomnia, and anxiety, which subsequently limited his outdoor activities. He denied visual disturbances, headaches, or sphincter dysfunction. Social history was significant for a 20-year smoking history (20 cigarettes per day). He denied alcohol consumption, recreational drug use, or specific dietary restrictions. Family history was unremarkable for consanguinity or neurological disorders.

### Initial medical and psychiatric assessment

Initial evaluation by an internal medicine physician yielded no significant physical findings. A comprehensive diagnostic workup—comprising electroencephalography, vestibular function testing, transcranial Doppler ultrasound, and computed tomography of the brain—was unremarkable. Serum B12 levels were not assessed at this stage. Given the negative workup and prominent affective symptoms, the patient was diagnosed with anxiety with somatization and initiated on venlafaxine hydrochloride (75 mg per day).

### Clinical progression and medication intolerance

Twenty days into the therapeutic regimen, the patient discontinued venlafaxine following the onset of intolerable epigastric pain. He was subsequently admitted to the gastroenterology department. An endoscopy revealed chronic non-atrophic gastritis with gastric erosion and bile reflux, while an abdominal CT scan showed no abnormalities. Concurrently, laboratory tests identified an abnormal serum vitamin B12 level (89 pmol/L; reference range: 133–675 pmol/L). However, tests for intrinsic factor and anti-parietal cell antibodies were negative, and levels of albumin, electrolytes, homocysteine and methylmalonic acid (MMA) were within normal limits. As a result, pernicious anemia was ruled out, and the deficiency was attributed solely to the patient’s recent reduced dietary intake. Despite treatment with omeprazole (40 mg per day), his somatic burden worsened, manifesting as chest tightness, memory decline, psychomotor slowing, and exacerbated nervousness.

### Psychiatric re-evaluation and neurological decline

Consequently, he was referred to the psychiatric department, where the diagnosis of severe anxiety and Somatic Symptom Disorder was reinforced. The treatment regimen was adjusted to include olanzapine, duloxetine, and clonazepam. Although this regimen provided transient stabilization of dizziness and mood, the patient subsequently developed progressive neurological deficits, including lower limb numbness, weakness, and gait instability, eventually resulting in occupational disability.

### Tertiary center evaluation and treatment

Nine months post-symptom onset, the patient was referred to our tertiary medical center. A comprehensive review of the clinical history, symptom evolution, and prior therapeutic responses was conducted (**Figure 1**), followed by a detailed multidisciplinary assessment:

**Figure 1:**
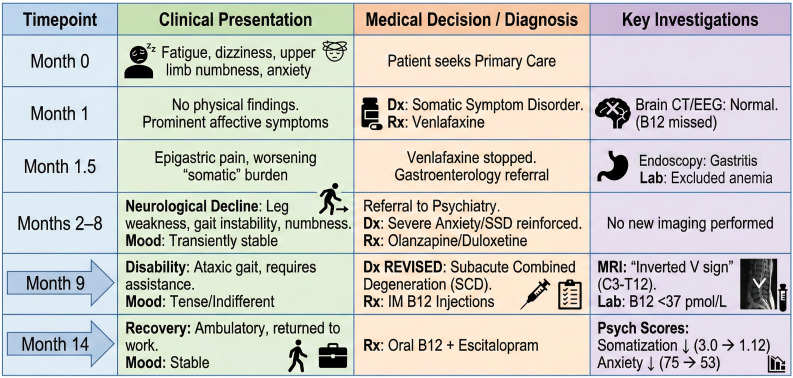
Timeline of clinical course and diagnostic evolution. The timeline illustrates the 14-month trajectory from symptom onset to recovery. Phase 1 (Months 0–8) represents the period of “diagnostic overshadowing,” where progressive neurological deficits were masked by psychiatric labeling and negative initial brain imaging. Phase 2 (Month 9) marks the tertiary center evaluation, identifying the characteristic radiological (“Inverted V” sign) and serological (B12 <37 pmol/L) markers of SCD. Phase 3 (Month 14) demonstrates functional recovery and the normalization of psychometric scores following targeted B12 replacement. Dx, Diagnosis; Rx, Treatment; SSD, Somatic Symptom Disorder; SCD, Subacute Combined Degeneration; CT, Computed Tomography; EEG, Electroencephalography; MRI, Magnetic Resonance Imaging.

#### Mental status examination

The patient was oriented but easily distracted. His speech was soft and decreased in spontaneity. Mood was tense with excessive worry, though he described emotional indifference rather than profound depression. Thought processes were slowed, with simplistic responses. Insight was preserved regarding his physical state, but he exhibited significant somatic preoccupation.

#### Neurological examination

Cranial nerves were intact, and fundoscopic examination was normal. Motor assessment demonstrated diffuse weakness in the lower extremities (grade 4/5). Deep tendon reflexes were hypoactive in the upper limbs, with bilateral patellar areflexia. Sensory testing indicated impaired vibratory, light touch, and pinprick sensation in the lower limbs, while joint position sense (proprioception) was preserved. The patient exhibited a wide-based, ataxic gait and required assistance for ambulation.

#### Psychometric assessment

Evaluation using the Self-Rating Depression Scale (SDS) and Self-Rating Anxiety Scale (SAS) yielded scores of 58 and 75, indicating mild depression and severe anxiety, respectively. Furthermore, the Symptom Checklist-90 (SCL-90) demonstrated a prominently elevated somatization subscale score (3.00), accompanied by increased scores in the depression (2.38) and hostility (2.33) dimensions.

### Investigations and diagnosis

Magnetic resonance imaging (MRI) of the cervical and thoracic spine was performed using a 3.0-Tesla scanner (MAGNETOM Prisma, Germany). The imaging protocol included sagittal and axial T1-weighted and T2-weighted sequences with a slice thickness of 3.0 mm. The T2-weighted sequences revealed extending hyperintense lesions from C3 to T12 in the posterior columns, exhibiting the characteristic “inverted V” sign on axial views (**Figure 2**). Laboratory analysis identified a critically low serum vitamin B12 level (<37 pmol/L; reference range 133–675 pmol/L). Electrophysiological testing showed mixed sensorimotor polyneuropathy. Nerve conduction studies demonstrated distal-predominant, symmetric axonal sensory involvement in both lower extremities, with demyelinating features in the motor nerves. Needle electromyography showed no spontaneous activity at rest, normal motor unit potentials with mild contraction, and a mixed recruitment pattern with maximal contraction. To exclude other etiologies, a brain MRI was performed and found to be normal. Comprehensive cerebrospinal fluid analysis, including a meningoencephalitis panel, alongside serum screening for infectious pathogens (including HIV), yielded negative results. Furthermore, an extensive autoimmune and rheumatologic workup-incorporating the antinuclear antibody spectrum and anti-myelin oligodendrocyte glycoprotein (MOG) antibodies-was entirely unremarkable. The diagnosis was confirmed as Subacute Combined Degeneration of the spinal cord secondary to vitamin B12 deficiency, complicated by comorbid anxiety.

**Figure 2:**
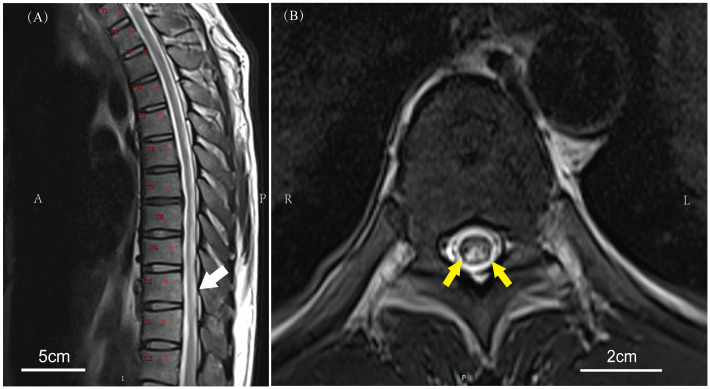
Magnetic resonance imaging findings characteristic of subacute combined degeneration. **(A)** Sagittal T2-weighted MRI revealing a longitudinally extensive hyperintense lesion involving the dorsal columns, extending from the C3 to T12 vertebral levels (white arrow). Orientation indicators: A (anterior), P (posterior). Scale bar: 5 cm. **(B)** Axial T2-weighted MRI of the spinal cord demonstrating symmetric hyperintense signal changes confined to the posterior columns (yellow arrows). This distinct pattern forms the characteristic “inverted V” sign for subacute combined degeneration. Orientation indicators: R (right), L (left), P (posterior). Scale bar: 2 cm.

### Treatment and outcome

The patient was treated with intramuscular vitamin B12 injections during hospitalization. Upon discharge, he was transitioned to oral vitamin B12 (1.5 mg/day) and prescribed escitalopram (10 mg/day) to manage the residual mood disturbance. At the five-month follow-up, the patient was fully ambulatory and had returned to work. Neurologically, patellar reflexes had returned, though mild vibratory sensory deficits persisted. Psychologically, significant improvement was observed: SDS scores decreased from 58 to 28, SAS scores decreased from 75 to 53, and the SCL-90 somatization factor dropped from 3.00 to 1.12.

## Discussion and conclusion

Subacute combined degeneration of the spinal cord is a treatable neurological complication of vitamin B12 deficiency, characterized by demyelination of the dorsal and lateral columns (10, 11). While its classic presentation involves sensory ataxia and pyramidal signs, early manifestations can be nonspecific, leading to diagnostic delays (13). The case presented here illustrates a potential instance of diagnostic overshadowing (9), where organic neurological symptoms were misattributed to a primary affective condition.

The potential misdiagnosis of SCD as a psychiatric disorder can complicate diagnosis and delay treatment. First, neurological recovery is time-sensitive, as prolonged demyelination can progress to irreversible axonal degeneration (7). In our patient, a nine-month delay likely contributed to the persistent of his gait instability. Second, inappropriate psychotropic medications can introduce side effects that may mask or exacerbate the underlying neurological symptoms (14). Third, as noted in previous literature, the psychological impact of having genuine physical symptoms dismissed as “psychogenic” may foster patient distress, erode the therapeutic alliance, and potentially amplify anxiety and somatization, creating a vicious cycle that further obscures the true diagnosis (15).

A critical aspect of this case was the interpretation of the patient’s distress. The patient initially presented with high scores on the SCL-90 somatization subscale, which previous clinicians interpreted as evidence of psychogenic illness. However, we propose that this interpretation may represent a diagnostic pitfall (16). In patients with active, undiagnosed neurological damage, high somatization scores might instead reflect genuine physical distress (e.g., paresthesia, weakness, sensory loss) rather than a primary psychiatric disorder. The “symptoms” recorded by the scale in this instance could be interpreted as neuropathic manifestations of dorsal column demyelination. Therefore, an elevated somatization score in the presence of subjective sensory complaints is perhaps best viewed not as definitive confirmation of a functional disorder, but rather as an indicator to rigorously exclude organic pathology.

The classic neuropsychiatric sequelae of vitamin B12 deficiency are frequently attributed to the accumulation of homocysteine and MMA, which disrupt neuronal membrane stability and induce metabolic neurotoxicity (17, 18). Notably, the patient in the present case exhibited profound neurological and psychiatric symptoms despite normal serum levels of homocysteine and MMA. While atypical, this phenomenon may suggest that localized central nervous system cobalamin depletion and early myelin instability may precede the systemic accumulation of these metabolic markers, particularly in cases driven by acute dietary restriction rather than chronic autoimmune pernicious anemia. This demyelination process might affect the spinal cord (frequently presenting as the characteristic “inverted V” sign on MRI) and the microstructure of cerebral white matter, which may lower the threshold for anxiety and mood instability (19, 20). Thus, this case may underscore the importance of integrating clinical findings, neuroimaging, and serum B12 levels for diagnosis, rather than relying exclusively on the presence of secondary metabolic markers.

This case highlights several “red flags” that might prompt clinicians to re-evaluate a psychiatric diagnosis: (1) Nature of Symptoms: The presence of specific neurological complaints—such as distal paresthesia (“pins and needles”) and gait instability—is highly unusual for purely psychogenic presentations. (2) Therapeutic Refractoriness: A discordance between therapeutic intervention and clinical trajectory can be an important indicator of a potential misdiagnosis (10). In this case, the progression of symptoms despite standard psychiatric treatment served as the crucial pivot point for diagnostic reframing. (3) Objective Signs: A meticulous neurological examination revealed absent knee jerks, impaired vibration sense, and a positive Romberg sign. These are objective neurological signs that are not adequately explained by anxiety or somatic symptom disorder alone.

Notably, this patient exhibited impaired vibratory sensation with preserved proprioception. Although both vibratory and proprioceptive (joint position) signals ascend via the posterior columns of the spinal cord, their conduction properties differ significantly (21). Vibratory sensation relies on high-frequency neural impulses, the transmission of which is highly dependent on the integrity of large myelinated fibers (22). Consistent with neurophysiological principles (22), the early demyelination induced by vitamin B12 deficiency disproportionately disrupts the conduction of these high-frequency action potentials. As a result, vibratory signaling is often among the first modalities to be compromised. Consequently, particularly in the early stages of SCD, patients might present with an isolated loss of vibration sense in the lower extremities, while low-frequency joint position sense remains temporarily intact. As demyelinating lesions progress, both sensory modalities typically become impaired.

The management of psychiatric symptoms in SCD should be guided by a comprehensive clinical assessment (23). While prompt treatment of the underlying organic etiology is the fundamental principle for managing secondary psychiatric syndromes (24)—with affective symptoms typically remitting as the primary disorder resolves—this trajectory was not fully realized in the present case. Following initiation of vitamin B12 supplementation (17), the patient’s neurological deficits, including ataxia and sensory impairment, improved; however, significant affective symptoms persisted. Given their impact on social functioning and their potential to interfere with adherence to treatment of the underlying condition (23), escitalopram (10 mg/day) was introduced as adjunctive therapy, with a favorable clinical response. Mechanistically, it is likely that B12 replacement halted the primary organic driver, while the selective serotonin reuptake inhibitor facilitated parallel neurochemical stabilization (25). This case may underscore the complexity of managing organic neuropsychiatric conditions: while the primary treatment must target the underlying etiology, symptomatic psychiatric management may serve as a valuable adjunct to alleviate patient suffering during the lengthy period of remyelination (5, 19).

## Conclusion

This case serves as a critical reminder that SCD can masquerade as a functional disorder. It highlights the clinical necessity of avoiding the premature labeling of persistent somatic complaints as “psychogenic” without a comprehensive exclusion of organic causes. Adherence to fundamental clinical principles-specifically, correlating subjective complaints with objective reflexes and sensory testing, and screening for metabolic markers like B12 and homocysteine-remains paramount in minimizing diagnostic errors and facilitating effective treatment.

## Data Availability

The raw data supporting the conclusions of this article will be made available by the authors, without undue reservation.
